# Management of Respiratory Failure Caused by COVID-19 after Thoracoscopic Esophagectomy

**DOI:** 10.6061/clinics/2021/e2483

**Published:** 2021-02-01

**Authors:** Flavio Roberto Takeda, Carlos de Almeida Obregon, Yasmin Peres Navarro, Marco Aurélio Santo Filho, Ulysses Ribeiro Junior, Rubens Antônio Aissar Sallum, Ivan Cecconello

**Affiliations:** Departamento de Gastroenterologia, Disciplina de Cirurgia do Aparelho Digestivo, Faculdade de Medicina FMUSP, Universidade de Sao Paulo, Sao Paulo, SP, BR

## BACKGROUND

Since December 2019, with the first descriptions of cases of pneumonia caused by the new coronavirus in Wuhan, China, the virus causing this infection has spread globally (1). One of the main characteristics of this new virus is its high transmissibility (2).

Since February 2020, when the first coronavirus disease 2019 (COVID-19) case was reported in Brazil, a series of changes have been incorporated for the treatment of cancer patients with COVID-19. In particular, for patients with esophageal cancer, additional precautions have been taken, as surgery for esophageal cancer alone has higher morbidity and mortality rates compared to that for other oncological surgeries (3). In this context, severe acute respiratory syndrome (SARS) caused by COVID-19 can pose a serious risk to the patient.

Patients with cancer are more susceptible to infection than individuals without cancer owing to their systemic immunosuppressive state caused by the malignancy and anticancer treatments such as chemotherapy or surgery. Therefore, these patients might have an increased risk of COVID-19 as well as a poorer prognosis (4).

We report the case of a young man who underwent thoracoscopic subtotal esophagectomy for distal esophageal adenocarcinoma who developed COVID-19 with severe clinical presentation.

## CASE PRESENTATION

A 34-year-old man was referred to our department in January 2020 after an incidental diagnosis of distal esophageal adenocarcinoma discovered during preoperative endoscopy for bariatric surgery. The patient was asymptomatic on admission.

Esophagogastroduodenoscopy revealed an ulcerated lesion 5 cm in length located 30 cm from the incisors. The biopsy confirmed the diagnosis of moderately differentiated adenocarcinoma with a MutS Homolog 2 (MSH2) mutation. Computed tomography (CT) of the chest and abdomen and whole-body positron emission tomography-computed tomography (PET-CT) revealed thickening of the middle/distal esophagus without other suspicious lesions.

After staging, preoperative chemoradiotherapy was started, similar to the CROSS trial (a platinum/taxane-based regimen associated with radiotherapy with a total dose of 41 Gy) (5). The treatment was administered between February and March 2020.

The re-staging exams showed a partial response to chemoradiotherapy, and subtotal esophagectomy was proposed. The procedure was performed on May 28, 2020, by thoracoscopy and laparoscopy without complications, except for a left pneumothorax secondary to the accidental opening of the pleural cavity that occurred during lymphadenectomy and was resolved with drainage.

During the first three postoperative days (PODs), the patient remained in the intensive care unit (ICU) without events and was discharged to the ward on POD 4, stable with no complaints. On the same day, both the thoracic and abdominal drains were removed. Chest radiography performed after drain removal showed adequate pulmonary expansion without any noticeable changes.

However, on POD 6, the patient had developed a low fever (100.2°F/37.8°C), with no other associated symptoms. Physical examination showed no changes and the cervical drain had a clear output. Blood cultures were collected and CT scans of the neck, chest, and abdomen showed no signs of fistula and small atelectasis in the lung bases. The patient had been using antibiotics (ceftriaxone and metronidazole) since the surgery, which were maintained at first.

**Figure 1:**
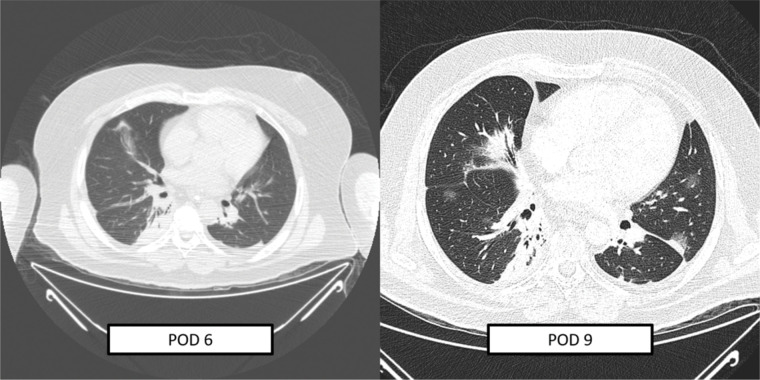
Evolutionary changes in thoracic computed tomography (CT) scans from the sixth to the ninth postoperative days (PODs).

The patient remained stable until POD 9 when he again developed a fever (100.4°F/38°C), associated with mild dyspnea and low peripheral oxygen saturation (88%). A new CT showed suspicious findings for viral pneumonia (ground-glass opacities and interlobular septal thickening). The antibiotics were replaced with piperacillin-tazobactam and the patient was referred to the ICU for follow-up care. Nasopharyngeal swabs were collected for severe acute respiratory syndrome coronavirus 2 (SARS-CoV-2) reverse transcription-polymerase chain reaction (RT-PCR), with positive results obtained the following day. Figure 1 shows the evolutionary changes in CT scans on PODs 6 and 9.

On the same day, he had also undergone a chest X-ray with oral contrast and methylene blue test, and no leaks were observed. He was receiving a pasty diet with good acceptance associated with enteral tube nutrition.

The fever, which was intermittent between POD 6 and POD 9, started to occur daily until POD 17.

In the following days, the patient developed progressive dyspnea requiring oxygen support, first by a common nasal cannula and then (by POD 14) by high-flow cannula and voluntary prone positioning. Despite these measures, the patient showed persisting unsatisfactory ventilatory parameters. After weighing the risks of dehiscence of the gastroesophageal anastomosis non-invasive ventilation was started. In making this decision, positive airway pressure was considered a potential benefit because the patient was obese with atelectasis on imaging examinations.

**Figure 2:**
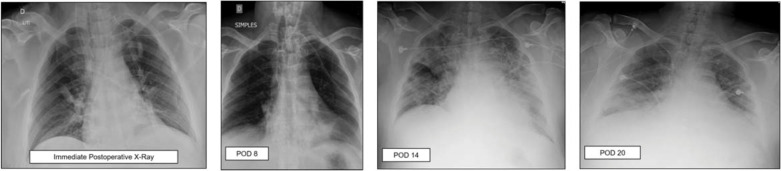
Evolution of chest X-rays over the days of hospitalization. POD=postoperative day.

**Figure 3:**
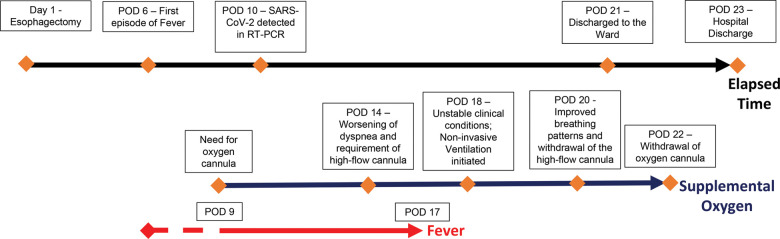
Timeline of the main events related to hospitalization. POD=postoperative day, SARS-CoV-2=severe acute respiratory syndrome coronavirus 2, RT-PCR=reverse transcription-polymerase chain reaction.

**Figure 4:**
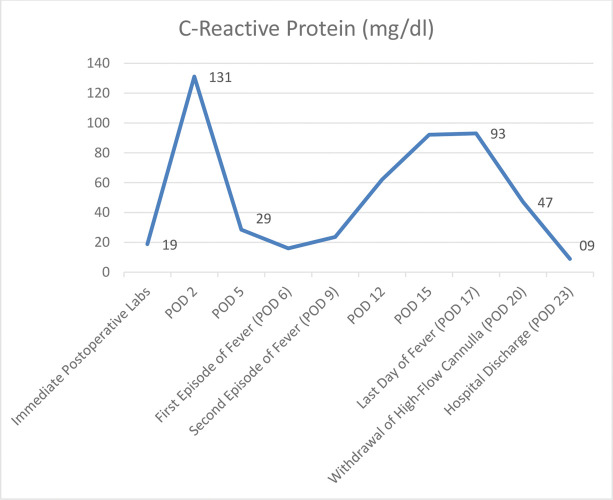
Variations in serum C-reactive protein levels during hospitalization. POD=postoperative day.

In the following days, a gradual improvement in respiratory parameters was noted, with radiographic improvement and the removal of the high-flow cannula on POD 20. The patient was transferred to the ward, where he remained for another 3 days. On POD 22, all supplementary oxygen was removed and he was discharged from the hospital on the next day. Figure 2 shows the evolution of chest X-rays over the days of hospitalization, Figure 3 outlines in chronological order the main events related to the patient’s evolution, and Figure 4 shows the variations of serum C-reactive protein (CRP) levels during hospitalization. Other laboratory findings did not vary as accurately as CRP.

## COMMENTS

There remains no prospect of epidemiological control of the COVID-19 pandemic in the coming months. Although clinical trials with vaccines are in advanced stages, these vaccines will not soon be accessible to the population. In this scenario, the social distancing of patients undergoing elective surgery as well as exams to screen for asymptomatic infection can help prevent perioperative contagion (6).

However, even with the adoption of preventive measures, it is still possible for false-negative patients to undergo surgeries or for intra-hospital transmission to occur (6). This occurrence is worrying as it can lead to much greater risks of respiratory complications and perioperative mortality (3,6).

The patient in the present case received tactics considered controversial for the postoperative period of esophagectomy, especially non-invasive ventilation. Non-invasive ventilation is usually contraindicated due to the potential risk of triggering the dehiscence of esophageal-gastric anastomosis (7). However, as there was a high risk of clinical worsening and the need for invasive mechanical ventilation, this alternative was reconsidered and precautionary measures were taken, such as limiting the positive pressure values in the ventilator.

We also questioned the possible role of the minimally invasive approach (thoracoscopy) when compared to open surgery in the evolution of this patient. The method adopted may have prevented worse outcomes, as it is less commonly associated with atelectasis and ventilatory restriction due to postoperative pain (8).

Health systems are still facing great difficulties in the management of cancer surgery during the current pandemic (4). Despite the fear of perioperative infection, many treatments cannot be postponed and such fatal complications may occur.

In these circumstances, it is important to adopt precautionary measures, such as careful consideration of symptoms, adoption of tests for diagnosis, and rapid isolation of the patient. Likewise, attention to the signs of clinical worsening and timely transfer to the ICU are important.
